# Treatment of oral fungal infections using photodynamic therapy: Systematic review and meta‐analysis

**DOI:** 10.1002/cre2.408

**Published:** 2021-04-02

**Authors:** Imaan Amina Roomaney, Haly Karen Holmes, Mark M. Engel

**Affiliations:** ^1^ Faculty of Dentistry, Department of Craniofacial Biology University of the Western Cape Tygerberg South Africa; ^2^ Faculty of Dentistry, Department of Oral Medicine & Periodontology University of the Western Cape Tygerberg South Africa; ^3^ Department of Medicine University of Cape Town Cape Town South Africa

**Keywords:** antifungal resistance, denture stomatitis, mycoses, photodynamic therapy

## Abstract

**Objectives:**

This systematic review evaluated the evidence for the effectiveness of Photodynamic therapy (PDT) in treating oral fungal infections, as an alternative to conventional antifungal medications.

**Methods:**

Five randomized control trials (168 participants) comparing the treatment of oral fungal infections using met with our inclusion criteria. Clinical and microbiological improvement was assessed by random‐effects meta‐analysis. Methodological quality assessment and heterogeneity were performed using peer‐reviewed criteria. PROSPERO registration: CRD42017076.

**Results:**

PDT showed statistically non‐significant increased clinical efficacy (risk ratio (RR) = 1.47 [95% confidence interval (CI), 0.68; 3.17]; three studies, *n* = 108 participants, *I* ^2^ = 50%) and mycological efficacy (mean difference (MD) = 0.54 [95%CI, −0.71; 1.79]; three studies, *n* = 100; *I* ^2^ = 39%) at 30 days, as compared with conventional antifungal therapy. Lack of standardization of treatment parameters and variability in the assessment of outcomes was observed across the studies. All included studies had a moderate to low risk of bias.

**Conclusions:**

PDT showed comparable effectiveness at treating oral fungal infections, particularly denture stomatitis. The small number of studies in this review, small sample size and variability of methods and outcome measures across studies, highlight the need for more standardized studies with longer follow‐up periods to enable recommendation of PDT as an alternative to conventional antifungal therapy.

## 1 INTRODUCTION

Human fungal infections are a growing public health concern, affecting more than 300 million people annually (Faini et al., 2015). Some of the most common fungal infections in humans affect the oral cavity and are seen in the critically ill, immune‐compromised in neonates, babies, and denture‐wearers (Armstrong‐James et al., 2014). They significantly impact the oral health‐related quality of life of the individual due to oral discomfort, burning, pain, dysgeusia (altered taste) and reduced appetite (Muzyka & Epifanio, 2013).

Treatment of oral fungal infections involves addressing predisposing factors (local and systemic) and pharmacotherapy. Topical antifungals are the first line of treatment for mucocutaneous fungal infections, followed by systemic antifungal medication (Muzyka & Epifanio, 2013). However, fungi are rapidly gaining resistance to currently available medication (Denning & Bromley, 2015; dos Santos Abrantes et al., 2014; Pfaller, 2012). In a recent study, 50% of *Candida albicans* specimens sampled were resistant to azoles (dos Santos Abrantes et al., 2014) and new drugs to treat fungal infections have not been developed since 2006 (Denning & Bromley, 2015). Alternative therapies are thus required to treat these minimally invasive fungal infections without propagating the rise in fungal antimicrobial resistance (Liang et al., 2016). Recently, the use of photodynamic therapy (PDT) has garnered attention as a potential antifungal treatment modality.

PDT, also referred to as photodynamic antimicrobial chemotherapy (PACT), photoradiation therapy and photochemotherapy, comprises three components: a chemical photosensitizer (PS), the application of light and the presence of oxygen. Briefly, the PS is applied to the target tissue (either topically or systemically). Light of an appropriate wavelength is then used to activate the PS, generating highly reactive oxygen species (ROS), including the singlet oxygen, in the target tissue. This results in cytotoxicity of the target cells and elicits an acute inflammatory response in the surrounding tissues (Konopka & Goslinski, 2007; Saini & Poh, 2013). Thus, PDT is being studied as a treatment modality for a variety of clinical applications, including the treatment of oral fungal infections; however, some recent studies have found PDT to be inferior when compared with antifungal medication in the treatment of specific oral fungal infections (Leite et al., 2015; Maciel et al., 2015). Given this equipoise, our systematic review sought to review current evidence on the use of PDT as a treatment modality for oral fungal infections in humans. In addition, we sought to determine the most effective treatment regimen parameters, light delivery parameters and which type and concentration of photosensitizers are most effective for the treatment of oral fungal infections. Lastly, we wished to determine how the risk factors for oral fungal infections such as smoking and diabetes mellitus, affect treatment outcomes.

## 2 MATERIALS AND METHODS

The protocol of this review was registered with PROSPERO, registration number CRD42017076421 and strictly complied with the Preferred Reporting Items for Systematic Review and Meta‐Analyses (PRISMA) guidelines (Moher et al., 2009). This review received an ethics waiver from the University of Cape Town, Faculty of Health Science Human Research and Ethics Committee as this review relied on only publicly available information (HREF 636/2018). No informed consent was required for this review.

### 2.1 Research question

This systematic review and meta‐analysis were conducted to address the following focused question: “Is photodynamic therapy compared with standard anti‐fungal treatment modalities, effective for the treatment of oral fungal infections in humans?”

### 2.2 Eligibility criteria

(a) Study design: Primary experimental and observational studies comparing the treatment of oral fungal infections using PDT to systemic and topical antifungal treatment were included; (b) Participants: Human participants with a clinical diagnosis and microbiological confirmation of an oral fungal infection; (c) Intervention: The use of PDT to treat an oral fungal infection in vivo; (d) Comparator: Any study using conventional topical or systemic antifungal medication for the treatment of oral fungal infections. We allowed flexibility with the antifungal drugs used and dosages of the comparator as treatment regimens vary in different settings and for different patients; (e) Outcome measures: The effectiveness of therapy was determined via clinical assessment and microbiological confirmation via direct microscopy or cell cultures. The presence or absence of *Candida* hyphae can be assessed and a change from hyphae present to absent would indicate improvement. Effectiveness was quantified by measuring the change in fungal load. The latter was quantified as *Candida* colony forming units per milliliter (CFU/mL). A decreased fungal load indicated an improvement in the condition. Semi‐quantification of CFU/mL is interpreted similarly; (f) Time frame and language: No restrictions.

### 2.3 Search strategy

A comprehensive database search was initially conducted in September 2018 using the following databases: The Cochrane Library, BioMed, SciELO, Scopus, EBSCOhost, PubMed/MEDLINE, ISI Web of Science, Clinicaltrials.gov, ProQuest, and WorldCat. The search strategy has been detailed elsewhere (Table S1; Roomaney et al., 2020). The results of the search were documented, reported and compared between databases (Table S2; Roomaney et al., 2020). The references were managed with EndNote (EndNote X9, version 9.2, Clarivate Analytics, USA) reference manager. An update on the search was conducted in PubMed on June 30, 2020.

### 2.4 Quality assessment

Each reviewer conducted an assessment of study quality and the risk of bias of each included study using the risk of bias tools of the Cochrane Collaboration (Higgins & Green, 2011).

### 2.5 Study selection and data extraction

The search results were collated within an online document and two researchers independently performed title and abstract screening, followed by full‐text evaluation and data extraction onto a pre‐design form. There was no disagreement between the reviewers on the studies to include.

### 2.6 Statistical analysis

Quantitative data was assessed using Review Manager (RevMan version 5.3) statistical software and the data were pooled, where appropriate, to conduct a meta‐analysis. Pooling of the data was done to assess three outcomes: (1) clinical improvement from baseline, (2) microbiological improvement by assessing changes in *Candida* colony forming units per milliliter (CFU/ml), and (3) microbiological improvement via semi‐quantification of CFU's. The studies assessed clinical change and microbiological change at different time points. Forest plots were created for the time points of 7, 15 and 30 days respectively. In conducting the meta‐analysis, we used the number of participants randomized to each arm, irrespective of withdrawal due to incompletion of treatment or loss to follow‐up, that is, intention‐to‐treat analyses.

The effect size was estimated and reported from continuous variables using mean difference and 95% confidence intervals. The weighting of each study was calculated using the inverse of the variance. A random‐effects model was used for analysis (Borenstein et al., 2010). Where the researchers found insufficient data, they conducted a narrative report of the results.

The authors used the Cochrane test (*P* < 0.1 cut‐off for statistical significance) to determine statistical homogeneity and the *I*^2^ test was used to quantify heterogeneity. The *I*^2^ test are interpreted as follows: 0%–40% may not be important; 30%–60% considered moderate heterogeneity; 50%–90% considered substantial heterogeneity; and 75%–100% is considerable heterogeneity (Higgins et al., 2019). Sub‐group analyses were planned to assess the following: the effect of different treatment parameters, including a comparison of different light delivery devices, wavelengths, photosensitizers and different treatment regimens i.e. duration of application, frequency of applications and time between applications; the various antifungal medications used; the effect of PDT on different fungal strains; and the effect of comorbidities/predisposing medical conditions such as HIV, diabetes mellitus, and dental prosthesis use. Publication bias was assessed via funnel plots, however, the low number of studies rendered them uninformative.

## 3 RESULTS

### 3.1 Literature search and study selection

**FIGURE 1:**
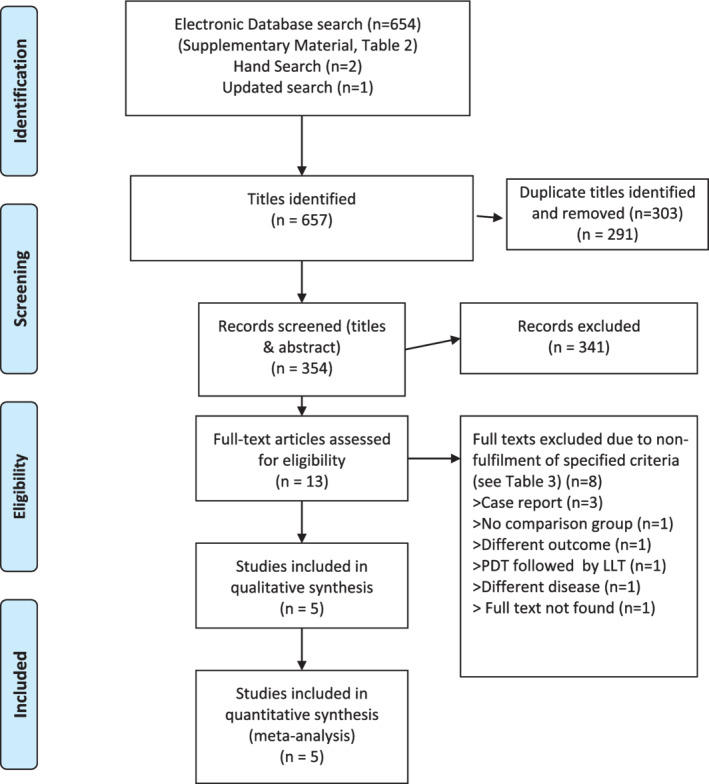
Schematic PRISMA flow diagram of the literature search. From: Moher, D., Liberati, A., Tetzlaff, J., Altman, D. G., The PRISMA Group. (2009). Preferred reporting items for systematic reviews and meta‐analyses: The PRISMA Statement. *PLoS Med*, *6*(7), e1000097. doi:10.1371/journal.pmed1000097

The initial search yielded 654 titles (Figure 1). Two additional articles were found by hand‐searching reference lists of relevant articles. Titles were collated and duplicates were excluded. The remaining 353 titles were evaluated, and 273 studies were excluded based on titles. Subsequent abstract screening resulted in a further 68 being excluded. We were unable to find the full‐text for one article (Cadastro & Giovani, 2009) leaving nine English language articles and two Portuguese language articles subjected to full‐text screening (Abduljabbar et al., 2017; Alves et al., 2018; Barcessat et al., 2017; Cadastro & Giovani, 2009; Maciel et al., 2016; Mima et al., 2011; Ribeiro et al., 2012; Simunovic‐Soskic et al., 2010). A further seven articles were excluded for not fulfilling the inclusion criteria. Details pertaining to the exclusion criteria are provided in the Supporting Information (Table S3; Roomaney et al., 2020). An updated search was conducted in June 2020 leading to the inclusion of an additional study (Alrabiah et al., 2019). Five full‐text studies were included in the review.

### 3.2 Characteristics of the included studies

The general characteristics of the included studies are presented in Table 1. Four of the randomized control trials included were conducted in Brazil (de Senna et al., 2018; Lopes, 2011; Mima et al., 2012; Scwingel et al., 2012) and one was conducted in Saudi Arabia (Alrabiah et al., 2019; *N* = 168). These studies comprise between 14 and 54 enrolled participants each.

**TABLE 1:** Characteristics of included studies

Study	Design	Population	Sample size	Mean age, male: Female	Follow up
Lopes, 2011	Randomized control trial	Patients presenting to the Dental Faculty at the University of São Paulo	Total: 24 12 individuals per arm	Not provided	30 days
Mima et al., 2012	Randomized control trial	Patients attending the Araraquara Dental School, Brazil	Total: 40 20 individuals per arm	Intervention: 62.45 (43–80) years 1:3 Control: 61.25 (41–78) 1:1.86	90 days
Scwingel et al., 2012	Three‐arm randomized control trial	Patients being seen by a customer Service Specialist at the City of Ponta Grossa (PR, Brazil)	Total: 14 7 individuals per arm	30 ± 8 years 3.2:1	30 days
de Senna et al., 2018	Experimental analytic randomized control trial with blinding	Patients presenting to the Odontology Faculty of Instituto Tocantinense Presidente Antonio Carlos in Araguaina, Brazil	Total: 54 27 individuals per arm	Overall: 56.4 years, 1:17 Intervention: 58.1 ± 6 years; 1:9 Controls: 54.7 years; ±7 18 females	30 days
Alrabiah et al., 2019	Randomized control trial	Not specified‐ Riyadh, Saudi Arabia	Total: 36 18 individuals per arm	Not provided	60 days

The description of parameters investigated, and technical characteristics of the PDT used in the included studies are documented in Table 2. Three studies conducted PDT on both dentures and oral mucosa (Alrabiah et al., 2019; de Senna et al., 2018; Mima et al., 2012). One study evaluated the treatment of oral candidiasis in HIV positive patients (Scwingel et al., 2012), while the other four studies specifically evaluated the treatment of denture stomatitis (Alrabiah et al., 2019; de Senna et al., 2018; Lopes, 2011; Mima et al., 2012).

**TABLE 2:** Description of parameters investigated and technical characteristics of the photodynamic treatments used in the included studies

Study	Treatment arms	Condition treated	Photosensitizer and light source	PDT parameters	Clinical outcomes	Microbiological outcomes
Lopes, 2011	Control: 5 mL 100,000 IU topical Nystatin, six times a day for 2 weeks Intervention: PDT of the lesion	Denture stomatitis	0.005% methylene chloride (Methylene blue; Chimiolux, Hipopharma) Twin laser (Twin Flex Evolution—MM Optics Ltda, São. Carlos, Brazil)	*λ*: 660 nm Power: 40 mW Energy density: 120 J/cm^2^ Length of application: 2 min per point Pre‐irradiation time: 20 min Number of application points: varies according to extent of the lesion. On average 1 cm apart Number of applications: two (1 week apart)	Budt‐Jorgenson classification Time points: before treatment, 48 h after treatment	Quantification via counts of CFU's Species identification via germ tube, micro‐culture in fermented agar and fermentation and assimilation of carbohydrates Time points: after first application, after second application (1 week later) and after 1 month
Mima et al., 2012	Control: Nystatin topical nystatin oral suspension 100,000 IU. Swish it for 1 min, gargle, and then expectorate it four times daily for 15 days Intervention: PDT of the palate and maxillary denture	Denture stomatitis	Hematoporphyrin derivative (Photogem®) LED Ten LEDs uniformly distributed on a circular platform	*λ*: 440–460 nm Power: 260 mW Energy density: 122 J/cm^2^ Intensity: 102 mW/cm^2^ Length of application: 20 min Number of applications: six sessions—three times per week for 15 days	Clinical assessment of infection severity using Newton's classification of denture stomatitis Time points: 0, 15, 30, 60, 90 days	Candida colony counts from the palate and denture surfaces quantified as CFU/mL *Candida spp*. prevalence Time points: days 0, 15, 30, 60, 90
Scwingel et al., 2012	Control: (fluconazole 100 mg/day for 14 days) Intervention 1: light laser therapy (LLT) Intervention 2: PDT of lesion	Oral candidiasis	Methylene blue Twin Laser	*λ*: 660 nm Power: 30 mW Length of application: 10 s Pre‐irradiation time: 1 min Number of application points: 9 Number of applications: 1	Clinical efficacy‐changing signs and symptoms from baseline Time points measured: every 2 days	Semiquantification of CFU of *Candida* spp. Time points measured: 0, 7, 15, 30 days
de Senna et al., 2018	Control: miconazole oral gel three times a day for 4 weeks Intervention: PDT of mucosa and dentures	Denture stomatitis	Methylene blue Laser GaAlAs—Photon Lase III—DMC	*λ*: 660 nm Power: 100 mW Pre‐irradiation time: 10 min Energy density: 28 J/cm^2^ Length of application: 20 s Number of applications: 8 (twice a week for 4 weeks)	Clinical efficacy: Budtz‐Jorgensen classification Time points: before treatment and 48 h after the end of treatment	Microbiological efficacy: response was assessed by the proposed method by Olsen (1974) Time points: before treatment and 48 h after the end of treatment (after 4 weeks)
Alrabiah et al., 2019	Control: Nystatin oral suspension of 100,000 IU gargled for 60 s, four times a day for 2 weeks Intervention: PDT of the palatal mucosa and maxillary denture	Denture stomatitis	Methylene blue (450 μg/mL) GaA1As	*λ*: 660 nm Power: 100 mW Pre‐irradiation time: 10 min Energy density: 28 J/cm^2^ Number of applications: 8 (two sessions per week for 4 weeks)	None	Microbiological efficacy: *Candida* counts from palates and surfaces of dentures. Measured in CFU/mL Total percentage counts of *Candida* spp. per group. Time Points: 0, 15, 30, and 60 days

Four studies used lasers as the light source (660 nm wavelength). Twin lasers were used in two studies (Lopes, 2011; Senna, 2012) and a GaAIA (Gallium aluminum arsenide) laser was used in two studies (Alrabiah et al., 2019; Scwingel et al., 2012). The studies investigating lasers utilized methylene blue as the PS. A single study used a hematoporphyrin derivative as a PS, which was activated by an LED light of 440–460 nm wavelength (Mima et al., 2012). The power of the LED used was 260 mW, which is significantly higher than that provided by the lasers (100, 40, and 30 mW respectively). Pre‐irradiation time, which is the length of time between application of the PS and photoactivation, ranged from 1 to 20 min. The length application of the laser per point was between 10 s and 2 min. The length of application of the LED was 20 min. Treatment sessions varied from one session (Scwingel et al., 2012) to two sessions 1 week apart (Lopes, 2011); to six sessions over 15 days (Mima et al., 2012). The largest number of sessions were eight PDT sessions over 4 weeks (Alrabiah et al., 2019; de Senna et al., 2018).

Three studies used nystatin suspension as the comparator. One advised rinsing with 5 mL of 100,000 IU suspension six times a day for 2 weeks (Lopes, 2011) and the other two studies advised rinsing with the same dosage, four times daily for 2 weeks (Alrabiah et al., 2019; Mima et al., 2012). The study with HIV‐positive participants used 100 mg of fluconazole daily for 15 days (Scwingel et al., 2012). In the fifth study, miconazole gel was applied to the affected area three times daily for 4 weeks (de Senna et al., 2018).

### 3.3 Method of clinical and microbiological assessment

Four studies assessed clinical change using three methods. Two studies used the Budtz‐Jørgensen et al. (1988) method before treatment and 48 h after treatment (de Senna et al., 2018; Lopes, 2011). One study (Mima et al., 2012) used Newton's classification (Newton, 1962) and the other study used specified subjective comparisons from clinical baseline to assess clinical changes (Scwingel et al., 2012). This was done at baseline, end of treatment (day 15) and on follow‐up (days 30, 60 and 90). Quantification of colony‐forming units (CFUs) was used to assess the microbiological success of treatment in three studies (Alrabiah et al., 2019; Lopes, 2011; Mima et al., 2012). The remaining studies made use of semi‐quantification of CFU/mL (de Senna et al., 2018; Scwingel et al., 2012). This was either done via visual assessment of the medium turbidity (clear, mild or intense) of cell cultures in test tubes and then scored as low, medium or abundant growth of fungus accordingly (Scwingel et al., 2012). Alternatively, the CFUs were counted and expressed as degrees of density (de Senna et al., 2018).

### 3.4 Study outcomes

**FIGURE 2:**
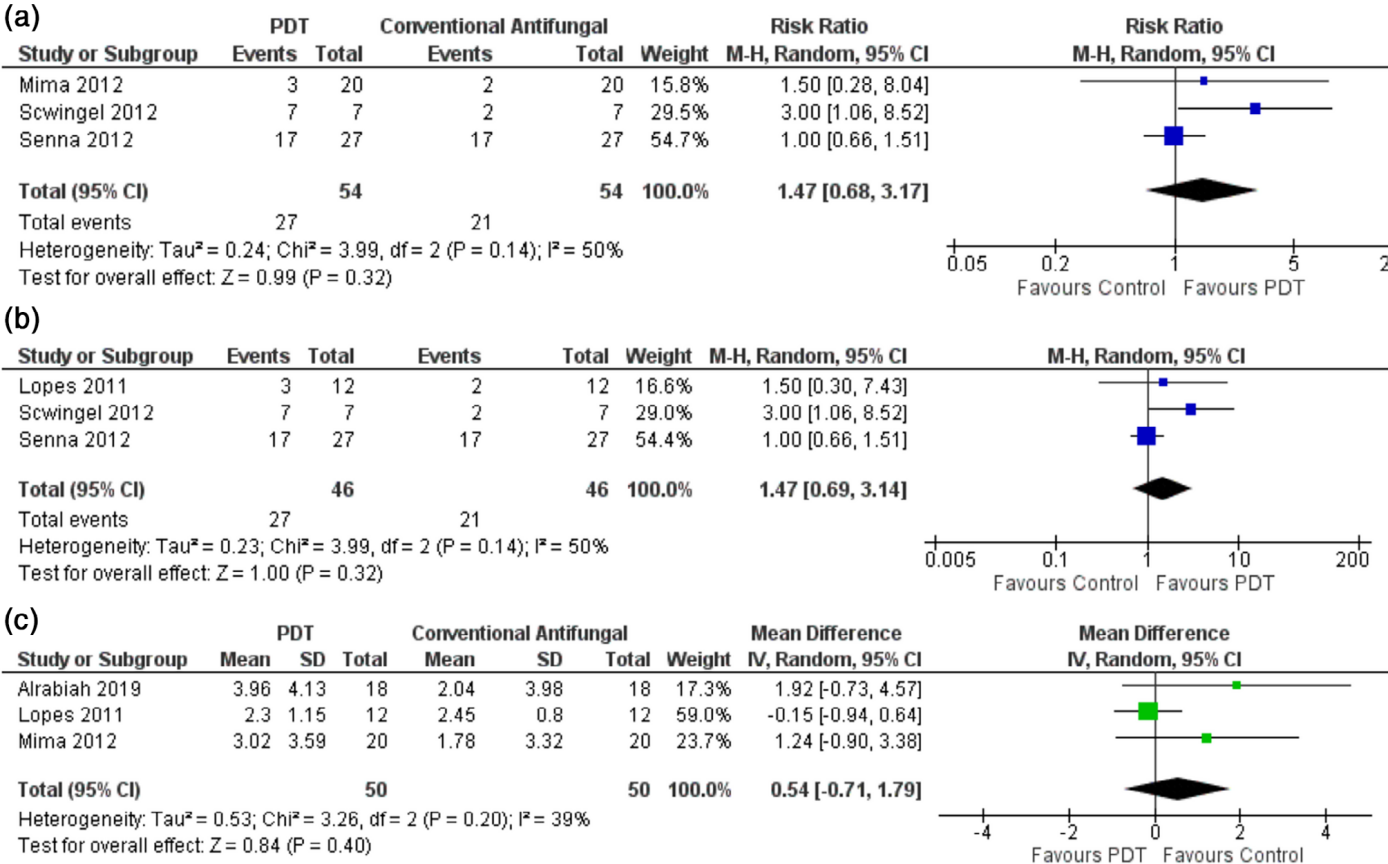
Forest plot of (a) clinical efficacy, (b) mycological efficacy using semi‐quantification of CFUs at 30 days, and (c) mycological efficacy using quantification of CFUs

There was no statistically significant difference in clinical effectiveness, that is, a reduction of oral lesions, between PDT and conventional antifungal therapy at 30 days (risk ratio (RR) = 1.47 [95% confidence interval (CI), 0.68; 3.17]; three studies, *n* = 108 participants; Figure 2a). Data for mycological efficacy assessed using semi‐quantification of CFUs supported these clinical findings (RR = 1.47 [95%CI, 0.69; 3.14]; three studies, *n* = 92; Figure 2b). Data for mycological efficacy assessed using CFUs showed no difference between the effectiveness of conventional (mean difference (MD) = 0.54 [95%CI, −0.71; 1.79]; three studies, *n* = 100; Figure 2c).

**FIGURE 3:**
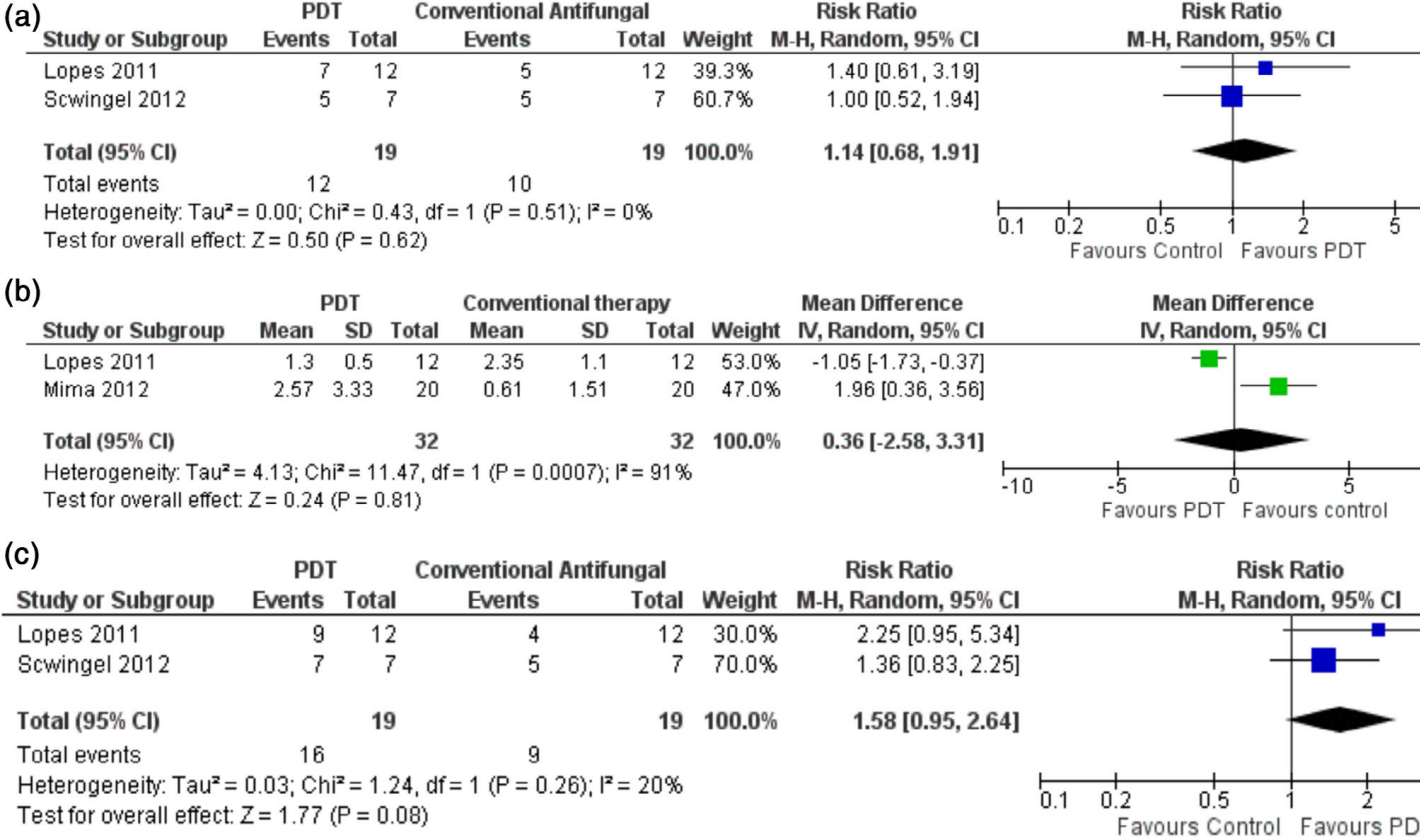
Forest plots of mycological effectiveness of treatment. (a) 7 Days using semi‐quantification of CFUs, (b) 15 days using quantification of CFUs, and (c) 15 days using semi‐quantification of CFUs

PDT showed similar mycological effectiveness compared to conventional medication, assessed at 7 days from the start of treatment (Figure 3a; RR = 1.14 ([95%CI 0.68; 1.91]; two studies; *n* = 38) and at 15 days using direct measurements (Figure 3b; MD = 0.36 [−2.58; 3.31]; two studies; *n* = 64) and indirect measurements (Figure 3c; RR = 1.58 [95%CI 0.95; 2.64], two studies; *n* = 38; Figure 3c). Extent of heterogeneity was low (*I* ^2^ = 20% (*p* = 0.26)) at 15 days and moderate (*I* ^2^ = 39% (*p* = 0.20)) at 30 days, which implies there was merit in pooling the data.

### 3.5 Risk of bias of included studies

Contact was made with authors to clarify the risk of bias of included studies. All included studies were found to have a moderate to low risk of bias (Table 3). Authors reported that due to the nature of the interventions, blinding of participants and personnel was not possible, however, all studies reported blinding of outcome assessors. Only one study reported allocation concealment (Alrabiah et al., 2019).

**TABLE 3:** Risk of bias of included studies

de Senna et al., 2018	Scwingel et al., 2012	Mima et al., 2012	Lopes, 2011	Alrabiah et al., 2019	
+^a^	+^a^	+^a^	+^a^	+^a^	Random sequence generation (selection bias)
−^b^	−^b^	−^b^	?^c^	+^a^	Allocation concealment (selection bias)
−^b^	−^b^	−^b^	−^b^	−^b^	Blinding participants and personnel (performance bias)
+^a^	+^a^	+^a^	+^a^	+^a^	Blinding of outcome assessment (detection bias)
+^a^	+^a^	+^a^	+^a^	+^a^	Incomplete outcome data(attrition bias)
+^a^	+^a^	+^a^	+^a^	+^a^	Selective reporting (reporting bias)
					Other bias

^a^ Positive (high quality).

^b^ Unclear/not fully fulfilled.

^c^ Negative (Low quality).

## 4 DISCUSSION

This systematic review and meta‐analysis found that photodynamic therapy (PDT) showed equivalent effectiveness in resolving oral fungal infections, however, to enable the recommendation of implementing PDT as an alternative management modality requires more studies with standardized methods and longer follow‐up periods.

This is the first systematic review and meta‐analysis performed on PDT and oral fungal infections analyzing only human studies. A concerted effort was undertaken to make the literature search thorough and comprehensive, limiting restrictions as much as possible. Authors were contacted to retrieve missing information. Despite finding only five studies meeting with our inclusion criteria, we were able to conduct a meta‐analysis to present aggregate data of PDT against conventional therapy.

Although the study designs of the included studies were similar, the studies demonstrated significant variability in their methods. The biggest challenges were the lack of standardization of treatment parameters across studies and inconsistency in the assessment of outcomes. Thus, in conducting the meta‐analysis, PDT was used as an umbrella term for any intervention (regardless of parameters) meeting our inclusion criterion. Three studies used the quantification of colony‐forming units (CFUs; Alrabiah et al., 2019; Lopes, 2011; Mima et al., 2012) as the outcome measure, as opposed to the semi‐quantification of CFU's used by the remaining two studies (de Senna et al., 2018; Scwingel et al., 2012). We conducted the planned subgroup analyses where data was available; however, the results were uninformative due to the small sample sizes after pooling of data. Although every effort has been made to reduce bias within our methods, these limitations should be considered when interpreting the results of this review.

Finding the most effective treatment parameters was a secondary objective as optimal treatment parameters of PDT has not yet been established in the literature. Treatment parameters varied significantly across the studies and may have influenced treatment outcomes. Since the optimal parameters of PDT are not yet established, these studies may be underestimating the effectiveness of PDT. Conversely, Antifungal medication used in the studies were used empirically, that is, the antimicrobial sensitivity of the fungi was not accounted for. Evidence exists that different fungal strains have variable sensitivity to the currently available antifungal medications (dos Santos Abrantes et al., 2014). Thus, this may skew data in favor of the PDT as the most appropriate antifungal medication and dosage may not have been used. Similarly, there is evidence that different fungal strains also have variable sensitivity to PDT (Dovigo et al., 2010). Alrabiah et al. (2019) and Mima et al. (2012) compared fungal species before and after treatment and had similar findings. *C*. *albicans* was similarly sensitive to PDT and nystatin (75% and 90% reduction), whereas, *C*. *tropicalis* appeared to be significantly more sensitive to nystatin than PDT at 15 days (45% and 50% reduction). A laboratory study by Dovigo et al. (2010) comparing the sensitivity of four fungal species to various PDT parameters found that *C*. *tropicalis* required PDT at a greater energy density for inactivation than that required by *C*. *albicans*. More research is required to confirm the clinical implication of the variable sensitivities of different fungal species to PDT and this should be considered when designing future studies. Furthermore, four of the studies focused on denture stomatitis. Other forms of oral fungal infections are not adequately represented. Future studies should focus on isolating fungal species, determining antimicrobial sensitivity and broadening the array of diseases being treated.

The few studies in our review precluded an assessment of the effect of local risk factors (such as smoking, nocturnal denture wearing, denture hygiene) and systemic risk factors such as HIV, Diabetes mellitus and immunosuppressive therapies, on oral fungal infection treatment outcomes. Smoking is a risk factor for oral fungal infections and treatment outcomes tend to be inferior in smokers compared to non‐smokers (Abduljabbar et al., 2017). The study by de Senna et al. (2018) which included four smokers found that miconazole was more effective at reducing fungal load than PDT. There was, however, only one smoker in the miconazole control group compared to three in the PDT group. Although the statistical significance of this finding was not mentioned, it is important to note that all smokers in the study presented with higher fungal loads at follow‐up. One study compared fluconazole treatment and PDT in HIV‐positive patients (Scwingel et al., 2012) but there was no comparison between the response between HIV‐positive and immunocompetent individuals to determine if their response was different. The remainder of the studies excluded patients with systemic risk factors which limits the evidence for the use on PDT in patients with systemic conditions and it is well established that those who are immune‐compromised are more likely to develop oral fungal infections. Therefore, it would be beneficial to include patients with systemic and local risk factors and report on their outcomes.

Recurrence of fungal infection has been mentioned as a particular concern when using PDT (Lopes, 2011; Mima et al., 2012). This corresponds to a recent case series treating five patients with denture stomatitis (Alves et al., 2018) which found recurrence in all patients at the end of day 45 of follow‐up. More studies beyond 30 days will be required to assess if recurrence is a problem with PDT therapy in general or to determine if it is related to the specific treatment parameters used. If recurrence is found to be a problem with the use of PDT, it would be important to assess whether new fungal species have emerged, PDT‐resistant species have developed or whether an insufficient reduction of patient risk factors is a possible contributory factor to the recurrence.

The importance of finding alternatives to conventional antimicrobial medication cannot be stressed enough. PDT appears to have potential as a therapy for oral fungal infections. However, the lack of recent human studies begs to question as to why progress into this area has stalled. At present, it is still a relatively costly procedure requiring specialized equipment, not commonly available in general dental offices. However, there is an effort to create a more cost‐effective LED light source (Daly et al., 2017; Hempstead et al., 2015) which would make PDT more accessible and provide greater scope to evaluate its impact than is currently possible. Moreover, other than recurrence, no major adverse effects such as burning and pain, have been found with the use of PDT in the treatment of oral fungal infections in this review. There is no risk of drug interactions which is a considerable problem with some antifungal medications. While little risk related to the use of PDT has been reported, more clinical research is required on all aspects of PDT treatment parameters. There is a need for well‐designed clinical trials which use standardized and objective clinical and microbiological outcome measures and comparable treatment parameters to allow a more robust meta‐analysis to be conducted and clinical guidelines to be developed.

The findings of this review and meta‐analysis suggest that Photodynamic therapy (PDT) is as effective at treating oral fungal infections, particularly denture stomatitis, compared with conventional antifungal medications, however, too little is known about the treatment parameters to endorse its clinical use. These findings are limited by the small number of studies and sample sizes. This work emphasizes the importance of standardized methods in conducting trials of this nature, ensuring high‐quality research with low risk of bias, adequate sample sizes and longer follow‐up periods with adequate reporting of other risk factors, given that they may affect treatment outcomes.

## CONFLICT OF INTEREST

The authors have stated explicitly that there are no conflicts of interest in connection with this article.

## AUTHOR CONTRIBUTIONS

IR, HH and ME were jointly responsible for the conceptualization of the study. IR and HH performed the data extraction. IR and ME conducted the meta‐analysis. All authors performed editing and approved of the final manuscript.

## Supporting information


**Appendix** **1,** Table 1: Example of Search Strategy for photodynamic therapy for oral fungal infections
Appendix **1,** Table 2: Titles retrieved by electronic, manual, and reference searching
Appendix **1,** Table 3: Clinical studies using PDT to treat Oral Fungal Infections which have been excluded

## Data Availability

The data that support the findings of this study are openly available in FigShare at Engel, Mark; Roomaney, Imaan (2020): Supplemental Information: Treatment of oral fungal infections using antimicrobial photodynamic therapy: A systematic review and meta‐analysis. University of Cape Town. Journal contribution. https://doi.org/10.25375/uct.12529517.v1.
